# Learning How Drug Companies Promote Medicines in Nepal

**DOI:** 10.1371/journal.pmed.0020256

**Published:** 2005-08-30

**Authors:** Bishnu Rath Giri, P. Ravi Shankar

## Abstract

An educational initiative looks critically at the drug industry's promotional tactics.

Armed with the power to prescribe, doctors are in a position to flood their patients' bodies with potent medicines at a stroke of their pen—and they have been consistently wooed by the pharmaceutical industry.

Doctors in Nepal have started to become targets for the pharmaceutical industry's promotional activities. The mountainous, landlocked, developing country of Nepal, which has a population of about 23 million, has an annual per capita income, in terms of purchasing power parity, of about US$2,000 in urban areas and US$1,000 in rural areas. In Nepal, Western allopathic medicine coexists with traditional medical practices like Ayurveda, Tibetan medicine, and faith healing. Many rural areas lack access to modern health care, but Kathmandu and other cities are becoming booming markets for pharmaceuticals.

## Pharmaceutical Promotion and Its Impact in Nepal

The promotional activities of medical representatives (MRs, also known as drug company representatives), and other promotional activities by pharmaceutical companies, have a major influence on prescribing and on drug use by the general public all over the world [1]. The World Health Organisation's Department of Essential Drugs and Medicines Policy maintains a database of such promotional activities at http://www.drugpromo.info. In Nepal, common prescribing problems are polypharmacy, and overprescription of antibiotics and injections [2]. We are studying prescribing patterns in our hospital and have observed excessive use of expensive antibiotics, vitamins, and digestive and enzyme preparations. The preparations prescribed are those that are actively promoted by MRs. I (PRS) have sometimes found consultants recommending to the hospital Drug and Therapeutics Committee that drugs of doubtful efficacy be included in the hospital pharmacy, following visits by MRs.

Most hospitals in Nepal allow free access of MRs to doctors, and academic detailing is absent. MRs bring with them many creative ideas for drug promotion. I (BRG) observed this for the first time when I visited one of my professors. A signboard next to the door read “Doctor is IN-DIGENE” (Digene is a brand of antacid). After going inside, I noticed that there was at least one big poster promoting a pharmaceutical company on every wall. On the table was a beautifully handcrafted nameboard with the professor's name in golden letters. The side facing the professor had the brand name of a drug in equally stylish lettering.

Medical conferences in Nepal are strongly dominated by the pharmaceutical industry. Often, companies organize parties for doctors in which a continuing medical education topic is followed by a lavish cocktail dinner—but often the educational part is absent. One such party was recently organized by a manufacturer of suture material and was attended by almost all the consultants and medical officers of our hospital.

After doctors accept personal benefits from the industry, they might arguably feel an obligation to prescribe the promoted drug. There is certainly good evidence in the literature that such gifts from the industry are associated with changes in prescribing behavior (the evidence is collected at http://nofreelunch.org/requiredinfluence.htm). Pharmaceutical companies also sponsor the activities of medical students (such sponsoring can take the form of sports matches, publications, and parties). The industry expects a substantial return for every rupee spent on doctors and medical students.

Often, though, the cost of promotion is directly recovered from the patient. A lady I (BRG) knew once said to me, “I am taking only half the medicine. If it works well then I will get more. It is too expensive.” The tendency to buy a lesser amount of the drug or no drug at all is a common phenomenon in Nepal and leads to therapeutic failure.

## An Educational Initiative on Promotion

**Figure 1:**
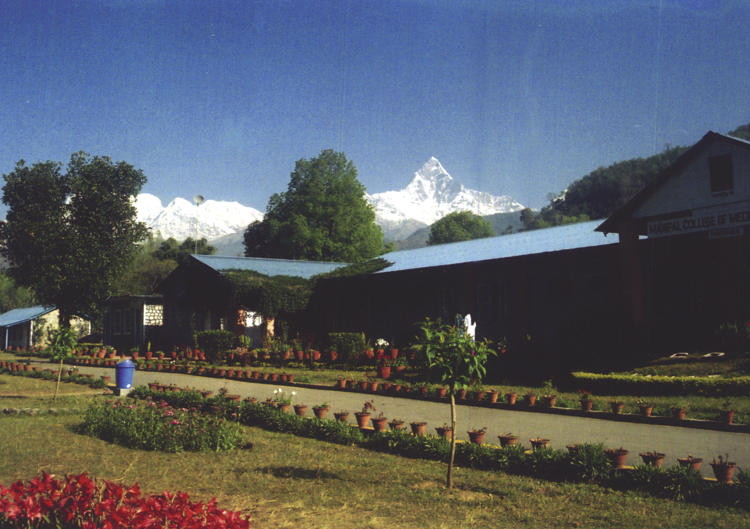
Manipal College of Medical Sciences, Pokhara, Nepal (Photo: Ravi Shankar)

As a student at Manipal College of Medical Sciences (Figure 1), I (BRG) have had the opportunity to experience an educational initiative on drug companies' promotional activities. This initiative involves small groups of students in interactive learning sessions. I have learned about common methods of drug promotion; unethical promotion practices; ways in which promotional materials give false impressions of the absolute and relative risks and the risk-benefit ratio; and the interpretation of graphs used in advertisements and optimizing time spent with MRs.

Class activities include critical analysis of promotional material and drug advertisements against the World Health Organization's Ethical Criteria for Medicinal Drug Promotion (http://www.who.int/medicines/library/dap/ethical-criteria/ethicalen.shtml). These criteria, prepared by an international group of experts, “constitute a frame of reference for judging proper behavior in drug promotion, whether involving the contents of advertisements and package inserts or the sponsorship of scientific symposia, and the use of representatives.” The criteria “give manufacturers, distributors, the promotion industry, prescribers and consumer groups a framework to ensure that promotional practices are in keeping with acceptable ethical standards.” Recently, role-play has been introduced into the class activities to present the interaction between a MR and a doctor. The interactions are critically analyzed by the groups. Students are assessed in critical analysis of promotional material during the pharmacology practical examinations.

Prescribers often do not realize the influence of promotional materials and activities [3]. In our hospital, through the Drug Information Center, drug bulletin, and other measures, we are sensitizing prescribers to critically evaluate industry sources of drug information. Recognizing that it is easier to inculcate good prescribing habits in future doctors, teaching and learning about rational use of medicines and medicinal drug promotion are emphasized in the medical students' pharmacology course.

## Impact of the Initiative

There has been a noticeable impact on the attitudes of medical students towards drug promotion. I (BRG) and my fellow students have been sensitized to the negative effects of aggressive promotion. In our hospital, aggressive promotion is evident but criticism is often difficult to make. When I discussed this essay with an intern, he responded, “No MR will approach you. You will need them from your internship onwards.”

I (PRS) am analyzing feedback obtained from a questionnaire about the educational initiatives. Preliminary assessment showed that students were favorably disposed toward and enjoyed the sessions. We are investing in the future, and believe that around 10%–15% of our students have been convinced to take a hard, critical look at drug promotion. “Enlightened” students will be an important asset to their communities and countries. Long-term studies of the impact will be carried out in the future.

## Conclusion

The initiatives have helped me (BRG) critically analyze sources of information from the pharmaceutical industry. In the future, I am confident that I will be prepared for the sales pitches and gimmicks of the industry.

When prescribers are made aware of the methods used by drug promoters, it is more difficult for unhealthy practices to take root; the ultimate beneficiaries are the patient and the community. We (PRS and colleagues) plan to bring in actual MRs to demonstrate the promotional techniques and to show videotaped encounters between doctors and MRs. We plan to strengthen the role-plays in the future. We are trying to enlist the support of the hospital's Drug and Therapeutics Committee to conduct sessions on drug promotion for the interns and medical officers.

**Writing for Student Forum**

If you would like to write an essay for the Student Forum, please send an enquiry to E-mail: studentforum@plos.org, stating in 100 words what the essay will be about and why it will be of interest to our readers. Further instructions on writing for the Student Forum, together with archived Student Forum articles, can be found at http://studentforum.plosmedicine.org. An international team of medical student advisers helps to choose articles for this section.

## Abbreviation

MR: medical representative
